# Supplementary material for: A metabolic interplay coordinated by HLX regulates myeloid differentiation and AML through partly overlapping pathways
Source: Nat Commun. 2018 Aug 6;9:3090. doi: 10.1038/s41467-018-05311-4 (PMC6078963; doi:10.1038/s41467-018-05311-4)
Supplement: Supplementary file 1 — Supplementary Information [file 41467_2018_5311_MOESM1_ESM.pdf]

A metabolic interplay coordinated by HLX regulates myeloid differentiation and AML through partly overlapping pathways

Piragyte et al.

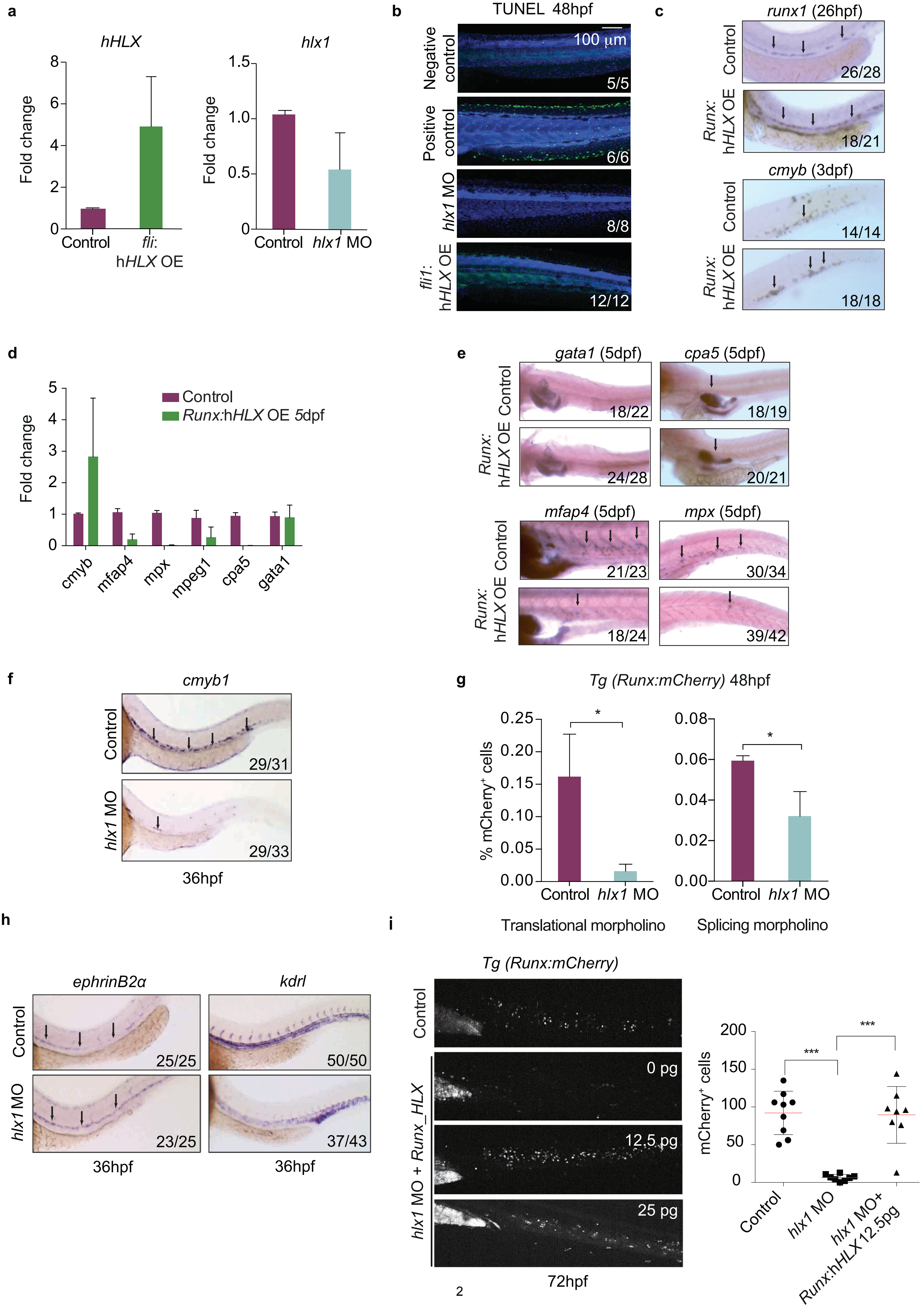
a
hHLX
hlx1
Fold change
Control
fli:
hHLX OE
hlx1 MO
b
TUNEL 48hpf
Negative control
Positive control
hlx1 MO
fli1: hHLX OE
100 μm
5/5
6/6
8/8
12/12
c
runx1 (26hpf)
Control
Runx: hHLX OE
26/28
18/21
cmyb (3dpf)
14/14
18/18
d
Control
Runx:hHLX OE 5dpf
cmyb
mfap4
mpx
mpeg1
cpa5
gata1
e
gata1 (5dpf)
cpa5 (5dpf)
18/22
18/19
24/28
20/21
mfap4 (5dpf)
mpx (5dpf)
21/23
30/34
18/24
39/42
f
cmyb1
29/31
29/33
36hpf
g
Tg (Runx:mCherry) 48hpf
% mCherry+ cells
Translational morpholino
Splicing morpholino
h
ephrinB2α
kdrl
25/25
50/50
23/25
37/43
i
Tg (Runx:mCherry)
hlx1 MO + Runx_HLX
0 pg
12.5 pg
25 pg
72hpf
mCherry+ cells
hlx1 MO+ Runx:hHLX 12.5pg

Supplementary Figure 1. *hlx1* regulates hematopoietic stem cell specification and definitive myelopoiesis in zebrafish

(**a**) qPCR analysis for *hlx1* expression in *fli*:h*HLX*OE endothelial cells and *hlx1*MO embryos (n=2: mean +s.d.). (**b**) TUNEL assay at 48hpf in *hlx1*MO or fli:h*HLX*OE embryos. The positive control was treated with DNase. Numbers in the bottom right corner of panels indicate numbers of zebrafish embryos with the indicated phenotype, versus the total number of zebrafish analyzed (n=3). (**c**) WISH for *runx1* at 26hpf and *c-myb* at 72hpf, in control or *Runx*:h*HLX*OE embryos. Numbers in the bottom right corner as described above. (**d**) qPCR results for *c-myb* and terminal myeloid markers as indicated, in control and *Runx*:h*HLX*OE whole embryos at 5dpf. (n=2, mean +s.d.). (**e**) WISH for terminal myeloid markers as indicated, in control or *Runx*:h*HLX*OE embryos at 5dpf. Numbers in the bottom right corner as described above. (**f**) WISH for *c-myb*, in control or *hlx1*MO embryos at 36hpf. Numbers in the bottom right corner as described above. (**g**) Graphs depicting the percentage of Runx$^+$ HSPCs in control *Tg(Runx:mCherry)* or *hlx1*MO injected with either a translational or a splicing morpholino at 48hpf measured by flow cytometry (n=3; mean + s.d.; Student's t-test, *P<0.05). (**h**) WISH for *ephrinB2α* (arterial endothelium) and *kdrl* (vascular endothelium) in control or *hlx1* MO embryos at 36hpf. Numbers in the bottom right corner as described above. (**i**) Representative images of *Tg(Runx:mCherry)* embryos at 72hpf, injected with *hlx1* morpholino and the indicated amounts of *Runx:*h*HLX* construct. The numbers of mCherry-positive HSPCs from each embryo are represented in the graph (mean + s.d., ANOVA test, ***P<0.001).

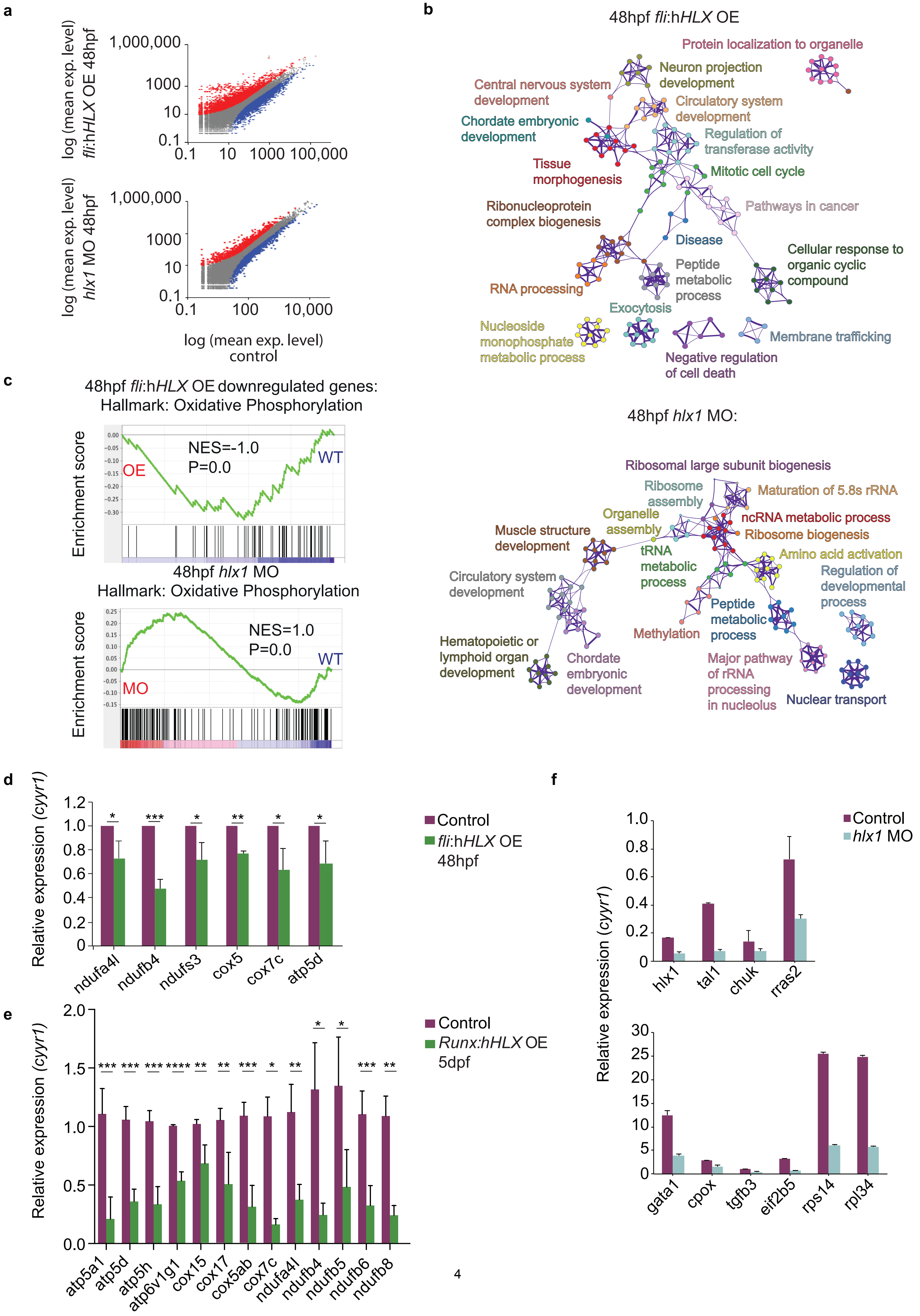
a
log (mean exp. level) fli:hHLX OE 48hpf
1,000,000
1000
10
0.1
0.1
10
1000
100,000
log (mean exp. level) hlx1 MO 48hpf
1,000,000
1000
10
0.1
0.1
100
10,000
log (mean exp. level) control
b
48hpf fli:hHLX OE
Protein localization to organelle
Neuron projection development
Central nervous system development
Circulatory system development
Chordate embryonic development
Regulation of transferase activity
Tissue morphogenesis
Mitotic cell cycle
Ribonucleoprotein complex biogenesis
Pathways in cancer
Disease
Cellular response to organic cyclic compound
Peptide metabolic process
RNA processing
Exocytosis
Nucleoside monophosphate metabolic process
Membrane trafficking
Negative regulation of cell death
48hpf hlx1 MO:
Ribosomal large subunit biogenesis
Ribosome assembly
Maturation of 5.8s rRNA
Organelle assembly
ncRNA metabolic process
Muscle structure development
Ribosome biogenesis
tRNA metabolic process
Amino acid activation
Circulatory system development
Regulation of developmental process
Peptide metabolic process
Methylation
Hematopoietic or lymphoid organ development
Chordate embryonic development
Major pathway of rRNA processing in nucleolus
Nuclear transport
c
48hpf fli:hHLX OE downregulated genes:
Hallmark: Oxidative Phosphorylation
Enrichment score
NES=-1.0
P=0.0
OE
WT
48hpf hlx1 MO
Hallmark: Oxidative Phosphorylation
Enrichment score
NES=1.0
P=0.0
MO
WT
d
Relative expression (cyyr1)
1.2
1.0
0.8
0.6
0.4
0.2
0
ndufa4l
ndufb4
ndufs3
cox5
cox7c
atp5d
Control
fli:hHLX OE 48hpf
e
Relative expression (cyyr1)
2.0
1.5
1.0
0.5
0.0
atp5a1
atp5d
atp5h
atp6v1g1
cox15
cox17
cox5ab
cox7c
ndufa4l
ndufb4
ndufb5
ndufb6
ndufb8
Control
Runx:hHLX OE 5dpf
f
Relative expression (cyyr1)
1.0
0.8
0.6
0.4
0.2
0
hlx1
tal1
chuk
rras2
Control
hlx1 MO
30
25
20
15
10
5
0
gata1
cpox
tgfb3
eif2b5
rps14
rpl34

Supplementary Figure 2. *hlx1* modulates the expression of metabolic genes in zebrafish endothelial cells and HSPCs

(**a**) Gene expression in control samples plotted versus gene expression in fli:h*HLX*OE (upper panel) or *hlx1*MO (lower panel) at 48hpf. Differentially expressed genes (>2 fold, NBT P<0.05) are coloured in red and blue for upregulated and downregulated genes, respectively. (**b**) Gene networks clustered on the basis of GO terms from the RNA-seq analysis of *fli*:h*HLX*OE and *hlx1*MO embryos. (**c**) GSEA analysis shows enrichement for oxidative phosphorylation in downregulated genes from *fli*:h*HLX*OE embryos and deregulated genes from *hlx1*MO. (**d-e**) qPCR validation of ETC genes in (**d**) *fli*:h*HLX*OE (48hpf) or (**e**) *Runx*:h*HLX*OE (5dpf) embryos (n=3 and n=4 respectively, mean + s.d.; Student's *t*-test, *P<0.05, **P<0.01, ***P<0.001, ****P<0.0001). (**f**) qPCR of selected genes from the RNA-seq data in *hlx1*MO (48hpf) (n=2, mean + s.d).

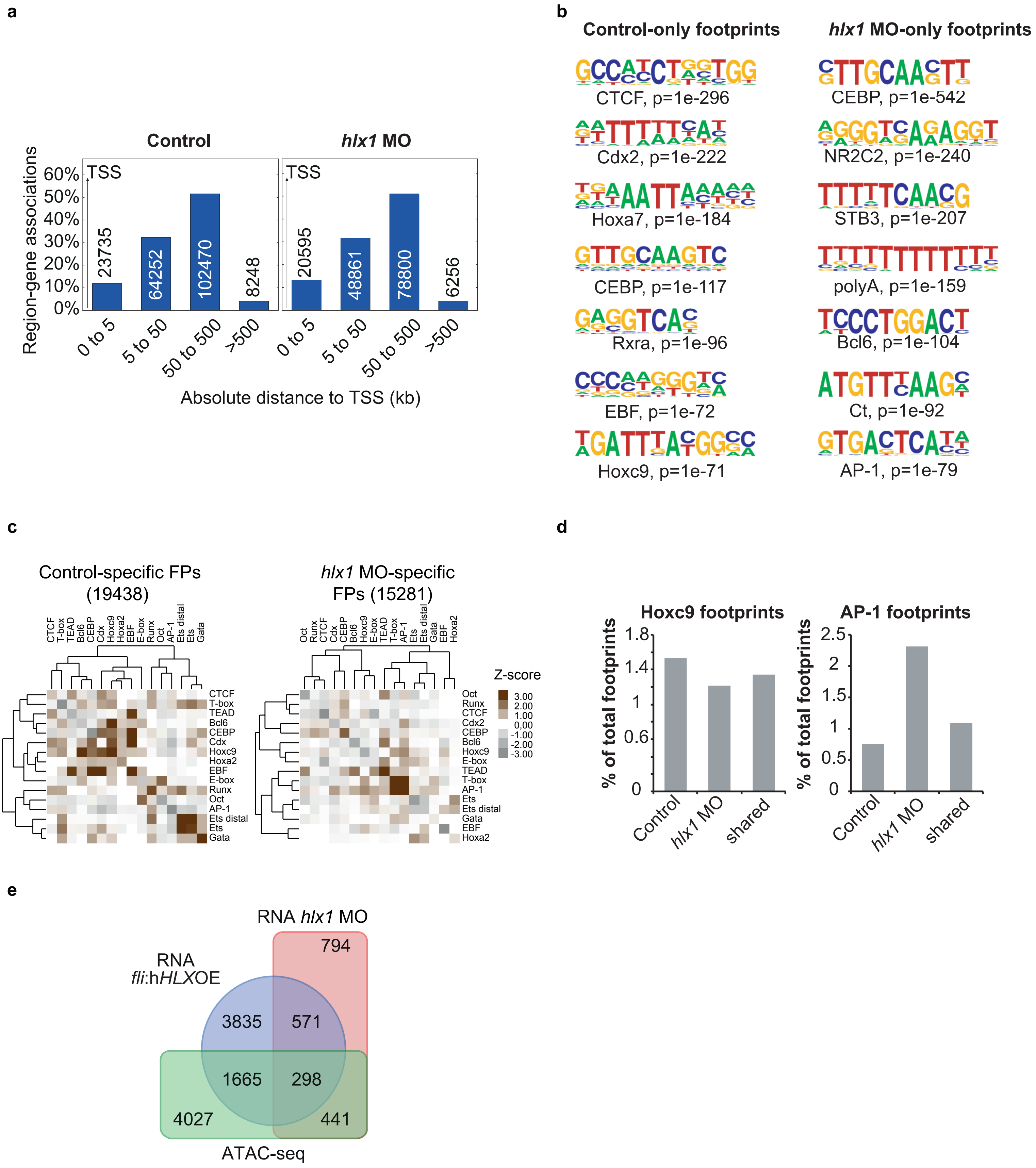

Supplementary Figure 3
Changes in chromatin accessibility in *hlx1* morphants
(**a**) GREAT analysis of the region to gene associations for the ATAC-seq peaks in control and *hlx1*MO. (**b**) Enriched motifs in control (left) specific or hlx1MO (right) specific footprints. (**c**) Hierarchical clustering of motif co-association enrichments in control (left) and *hlx1*MO footprints (right). z-scores represent enrichment of observed versus background self-enrichment and co-associations computed in randomly selected, equally sized total *hlx1* morphant and control footprints, respectively. (**d**) Bargraphs depicting the percentage of Hoxc9 (left) and AP-1 (right) motifs in control or *hlx1*MO specific footprints or in their shared footprints. (**e**) Venn diagram comparing differentially expressed genes in *hlx1*MO, *fli*:h*HLX*OE and differential ATAC-seq peaks in *hlx1*MO.

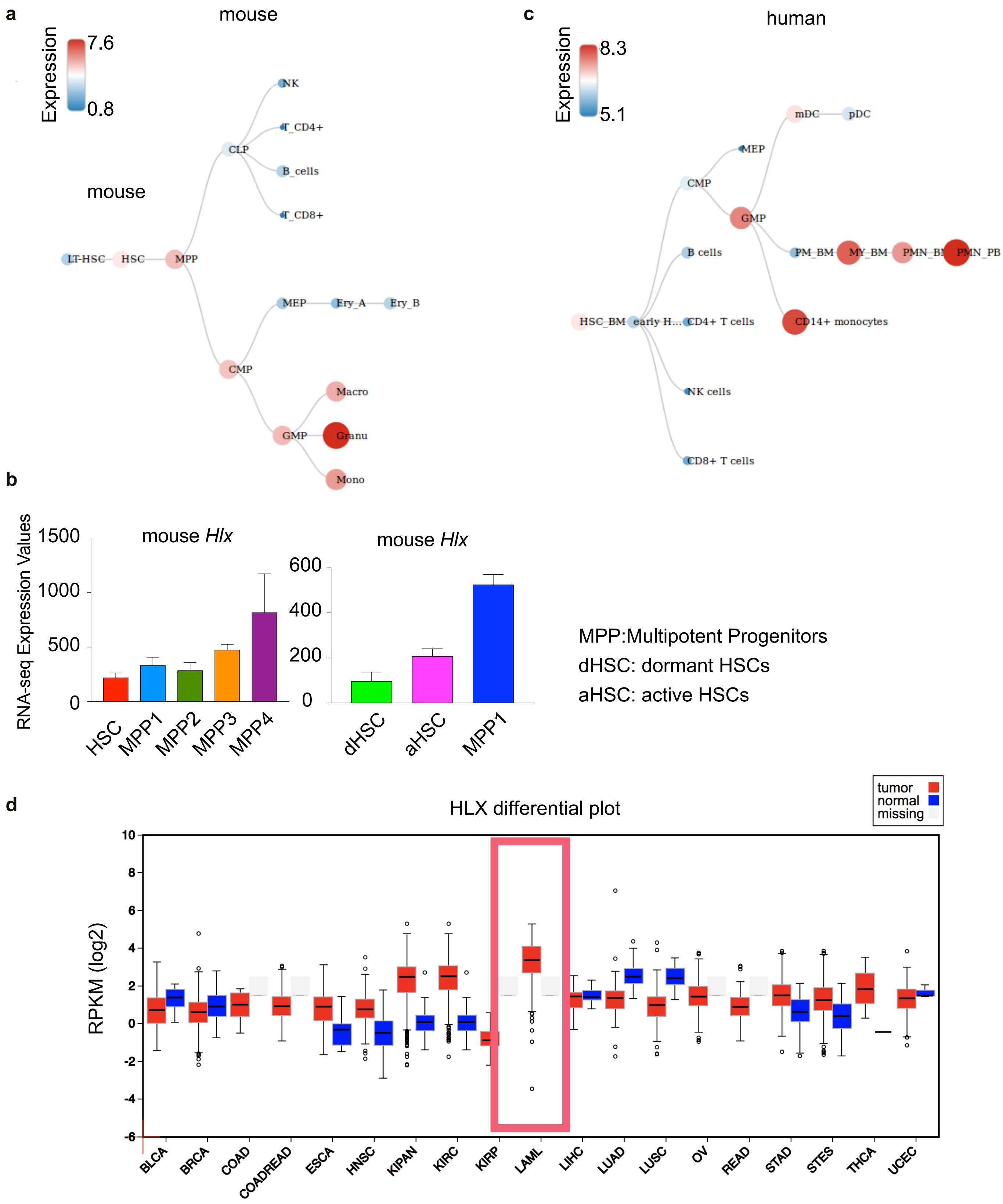

e

Canonical Pathways: patients' data	
Name	P-value
Oxidative Phosphorylation	0.0003
Mitochondrial Dysfunction	0.0107

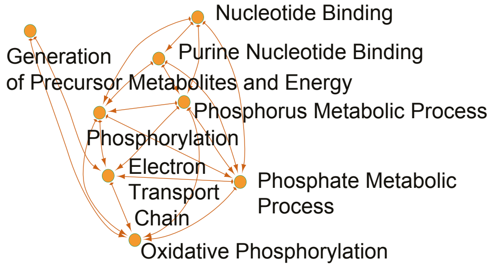

Supplementary Figure 4. HLX expression in murine and human hematopoietic populations

(**a**) *Hlx* expression in mouse hematopoietic populations. (**b**) *Hlx* expression in mouse HSPC populations (HSC, MPP1-4 as multipotent progenitors, dHSC as dormant HSCs and aHSC as active HSCs). (**c**) *HLX* expression in different human hematopoietic populations. (**d**) RPKM (log2) values for *HLX* expression in different cancerus samples from cBioportal/TCGA. (**e**) IPA analysis of genes from AML patients whose expression correlates with *HLX* expression. Gene network clustered on the basis of GO terms. Selected metabolic pathways are depicted (See also Supplementary Data 3).

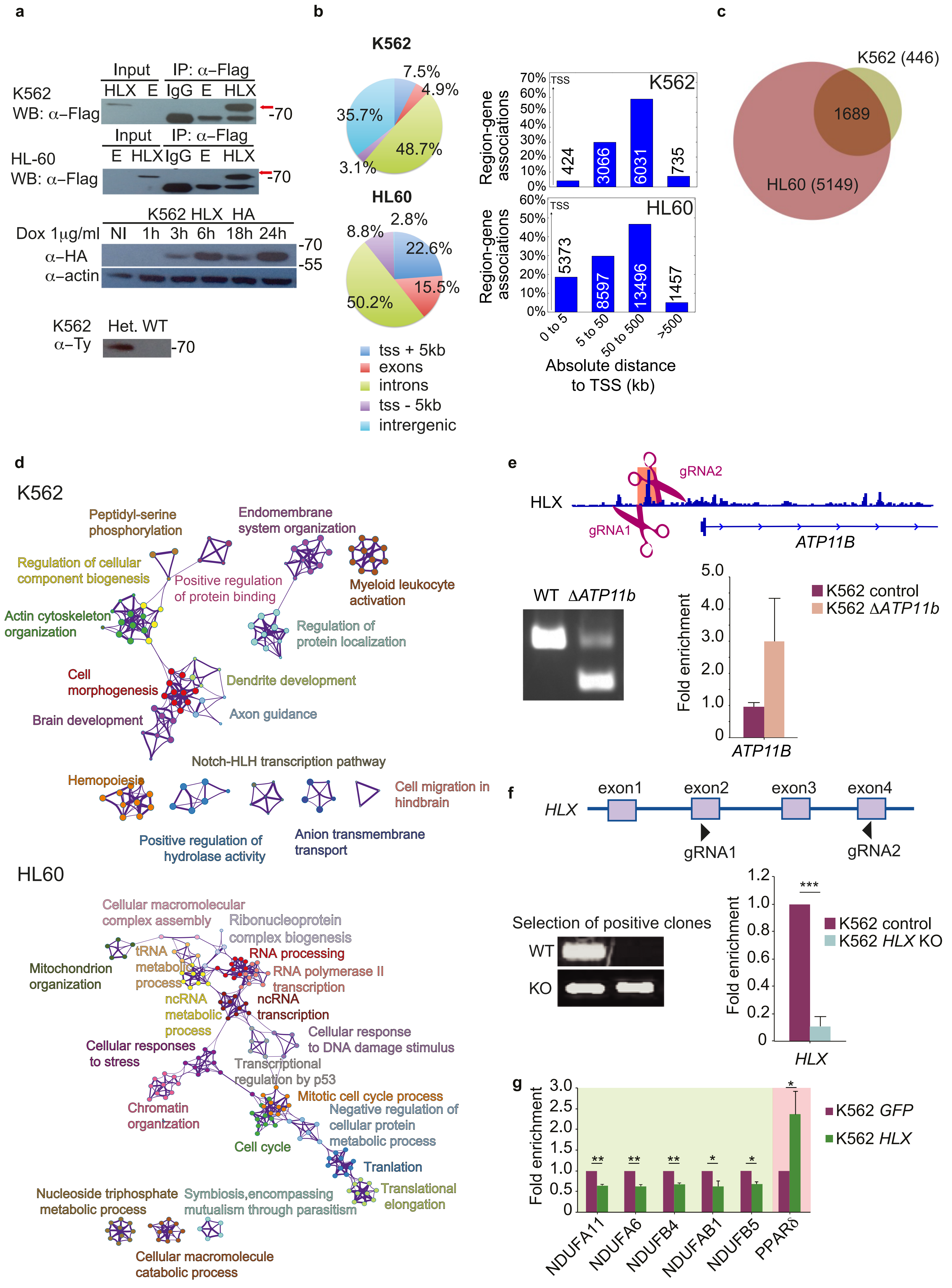

a
b
c
K562
WB: α-Flag
Input
IP: α-Flag
HLX E IgG E HLX
-70
HL-60
WB: α-Flag
E HLX IgG E HLX
-70
K562 HLX HA
Dox 1μg/ml NI 1h 3h 6h 18h 24h
α-HA
α-actin
-70
-55
K562 Het. WT
α-Ty
-70
K562
7.5%
4.9%
35.7%
48.7%
3.1%
HL60
2.8%
8.8%
22.6%
15.5%
50.2%
tss + 5kb
exons
introns
tss - 5kb
intrergenic
Region-gene associations
TSS
K562
424
3066
6031
735
HL60
5373
8597
13496
1457
0 to 5
5 to 50
50 to 500
>500
Absolute distance to TSS (kb)
K562 (446)
1689
HL60 (5149)
d
K562
Peptidyl-serine phosphorylation
Endomembrane system organization
Regulation of cellular component biogenesis
Positive regulation of protein binding
Myeloid leukocyte activation
Actin cytoskeleton organization
Regulation of protein localization
Cell morphogenesis
Dendrite development
Brain development
Axon guidance
Notch-HLH transcription pathway
Hemopoiesis
Cell migration in hindbrain
Positive regulation of hydrolase activity
Anion transmembrane transport
HL60
Cellular macromolecular complex assembly
Ribonucleoprotein complex biogenesis
Mitochondrion organization
tRNA metabolic process
RNA processing
RNA polymerase II transcription
ncRNA metabolic process
ncRNA transcription
Cellular responses to stress
Cellular response to DNA damage stimulus
Transcriptional regulation by p53
Chromatin organization
Mitotic cell cycle process
Negative regulation of cellular protein metabolic process
Cell cycle
Tranlation
Translational elongation
Nucleoside triphosphate metabolic process
Symbiosis,encompassing mutualism through parasitism
Cellular macromolecule catabolic process
e
HLX
gRNA2
gRNA1
ATP11B
WT ΔATP11b
Fold enrichment
K562 control
K562 ΔATP11b
ATP11B
f
HLX
exon1 exon2 exon3 exon4
gRNA1
gRNA2
Selection of positive clones
WT
KO
Fold enrichment
***
K562 control
K562 HLX KO
HLX
g
Fold enrichment
K562 GFP
K562 HLX
NDUFA11
NDUFA6
NDUFB4
NDUFAB1
NDUFB5
PPARδ

Supplementary Figure 5. HLX ChIP-seq analysis in HL60 and K562 cells

(**a**) Representative western blots of Flag-tagged, HA-tagged or 3xTy-tagged HLX (E: empty control, Dox: doxycyclin, WB: Western Blot, IP: Immunoprecipitation). Arrows show the band of Flag-HLX. **b**) Genomic distribution of HLX ChIP-seq peaks in K562 and HL-60 cells. GREAT analysis of region to gene associations in K562 and HL60 cells. (**c**) Venn diagram depicting the overlap of genes bound by HLX in K562 and HL60 cells. (**d**) Gene network clustered on basis of GO terms of HLX bound genes in K562 and HL60 cells. (**e**) Schematic depicting the strategy for generating deletion using CRISPR-Cas9 at the HLX bound region on *ATP11b* gene (Δ*ATP11B*) in K562 cells. Representative PCR genotyping of one heterozygote clone. qPCR analysis of *ATP11b* gene upon deletion of HLX bound region. (n=2; mean +s.d.) (**f**) Schematic depicting the strategy for generating knockout *HLX* clones with CRISPR-Cas9 system. Representative PCR genotyping of one heterozygote and one homozygote knockout clone. qPCR verification of *HLX* expression (n=3, mean + s.d., Student's *t*-test, ***P<0.001). (**g**) qPCR analysis of selected genes in K562 cells upon overexpression of HLX (n=3, mean + s.d., Student's *t*-test, *P<0.05, **P<0.01).

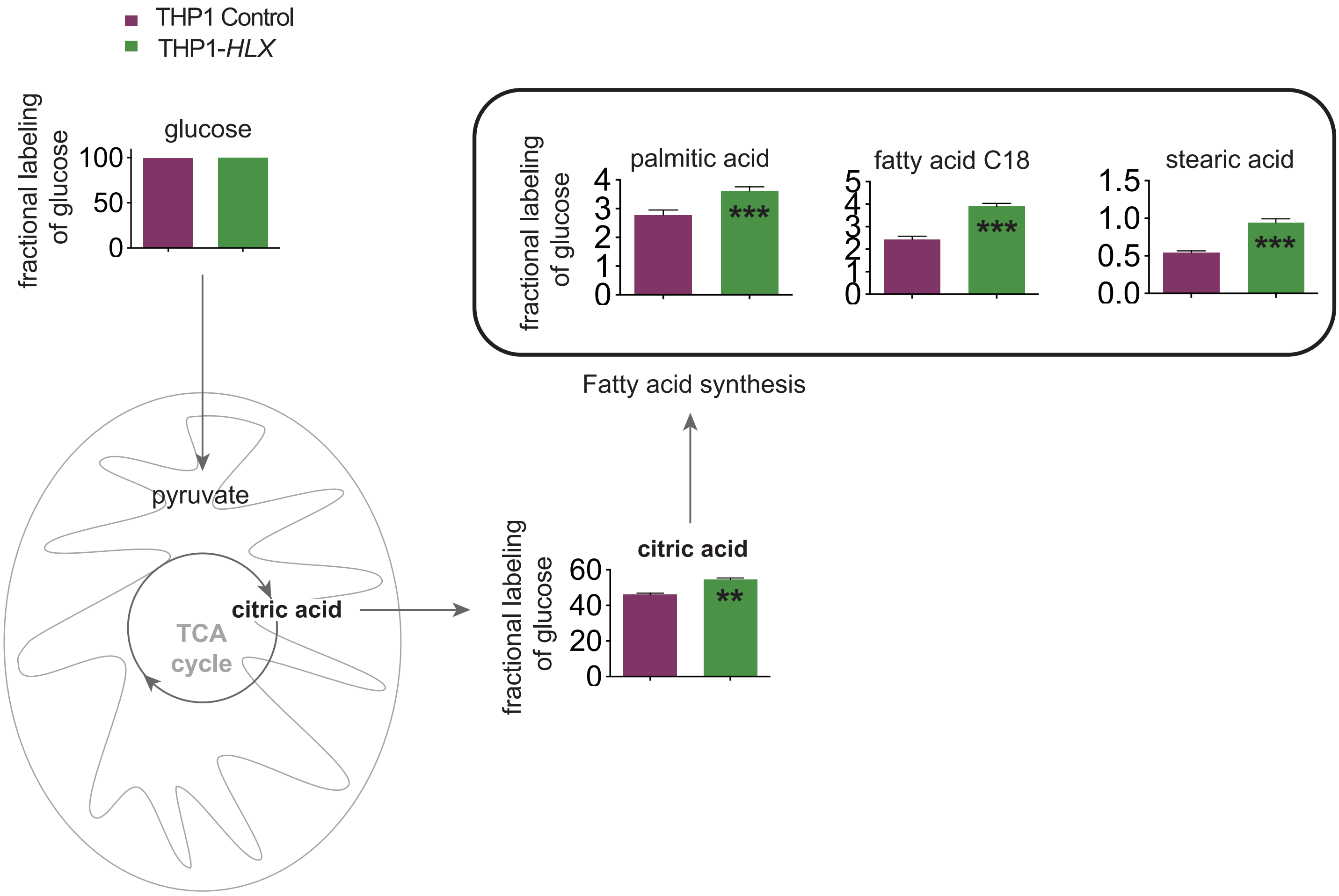

Supplementary Figure 6
Glucose tracing upon *HLX* overexpression

HLX overexpression in THP1 cells alters the allocation of Carbons from Glucose (Data shown as mean +/- s.d, representative of three samples per condition, Student's t-test, **P<0.01, ***P<0.001).

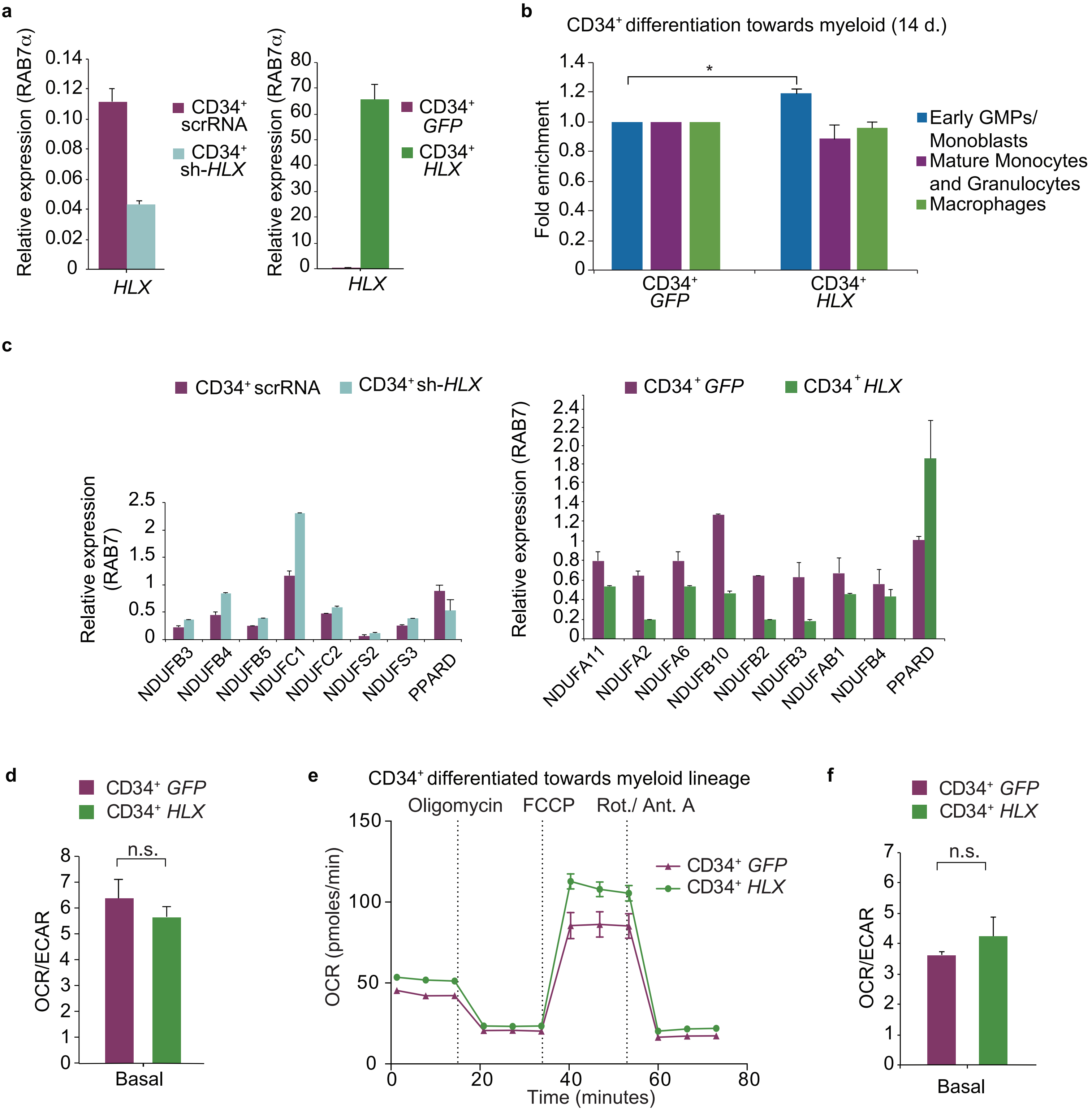

Supplementary Figure 7
Metabolic role of *HLX* in human CD34$^+$ cells

(**a**) Evaluation of the efficiency of HLX knockdown (CD34$^+$ scrRNA or sh-*HLX*) or HLX overexpression in CD34$^+$ (CD34$^+$ *GFP* or *HLX*) cells by qPCR (n=2, mean + s.d.) (**b**) Flow cytometric analysis of myeloid populations in CD34$^+$ *GFP* or *HLX* cells differentiated towards the myeloid lineage for 14 days. Change in cell populations represented as fold enrichment (n=3, mean + s.d., ANOVA test, *P<0.05). (**c**) qPCR validation of selected genes from the RNA-seq experiment upon *HLX* knockdown (upper panel) and *HLX* overexpression (lower panel) (n=2; mean + s.d.). (**d**) OCR/ECAR ratio of undifferentiated CD34$^+$ *GFP* or *HLX* cells (n=2, mean +s.d.). (**e**) Seahorse metabolic assay depicting OCR in CD34$^+$ *GFP* or *HLX* cells after differentiation towards myeloid cells for 14 days (n=2, mean + s.d.). (**f**) Representative OCR/ECAR ratio of CD34$^+$ *GFP* or *HLX* cells differentiated towards myeloid cells for 14 days (n=2, mean +s.d.).

All western blots in Figure 5d have the same order of samples as indicated in the first blot

Fig. 5d. HA-HLX

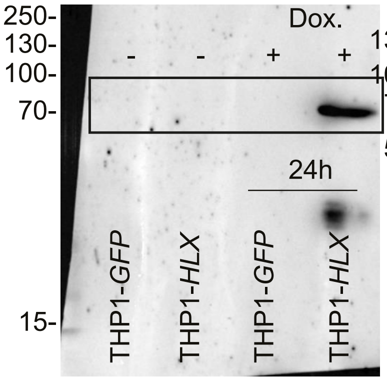

Fig. 5d. PPARδ

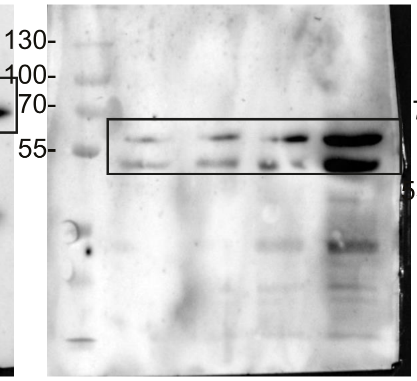

Fig. 5d. p-AMPK-α

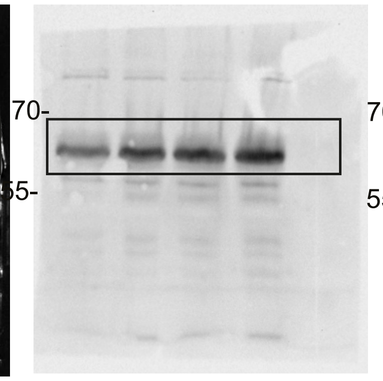

Fig. 5d. AMPK-α

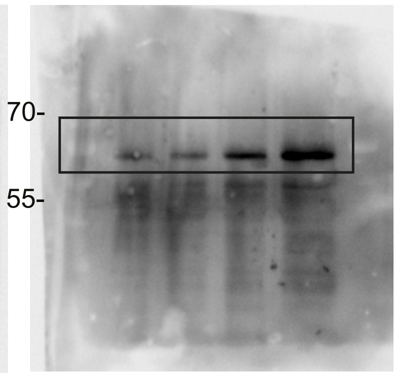

Fig. 5d. C-I, C-II, C-IV, C-V

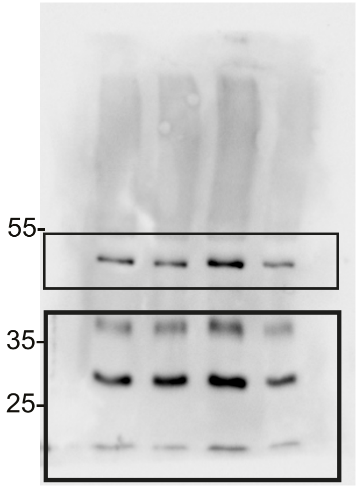

Fig. 5d. C-III

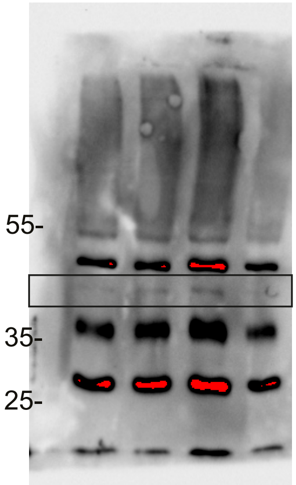

Fig. 5d. H3

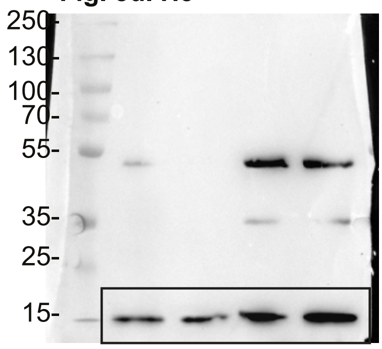

All western blots in Figure 5g have the same order of samples as indicated in the first blot

Fig. 5g. LC3-II

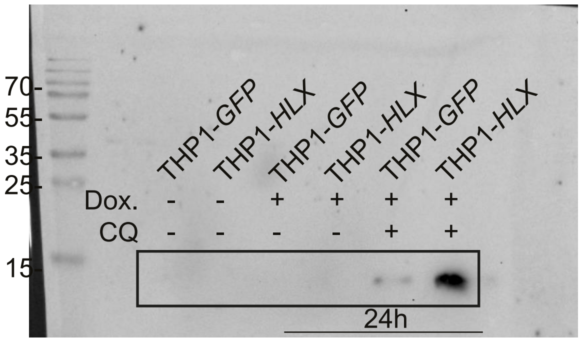

Fig. 5g. H3

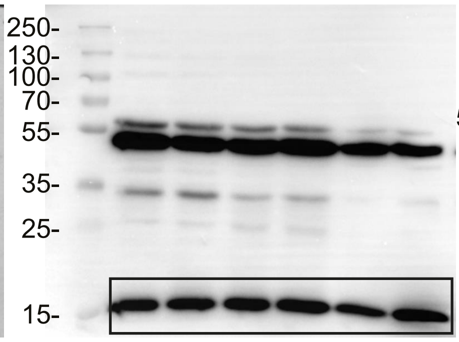

Fig. 5g. HA-HLX

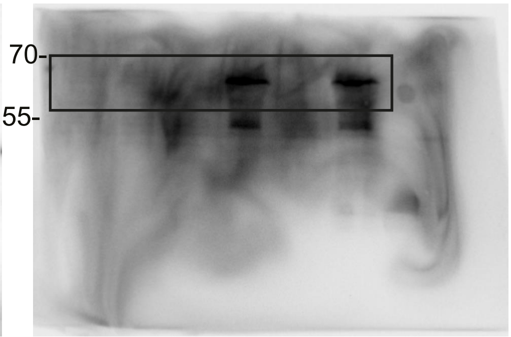

Supplementary Figure 8. Uncropped western blots

Fig. 8b. PPARδ

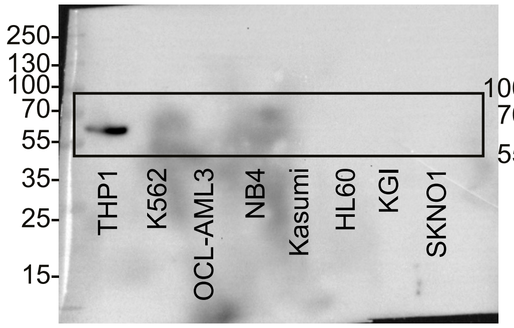

Fig. 8b. AMPK-α

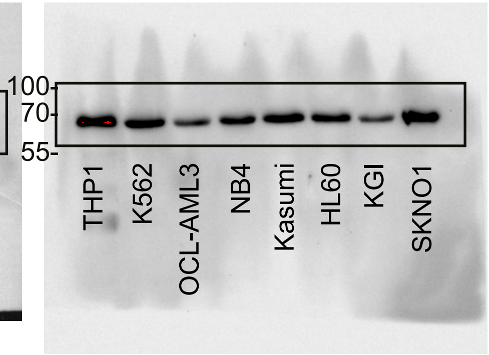

Fig. 8b. p-AMPK-α

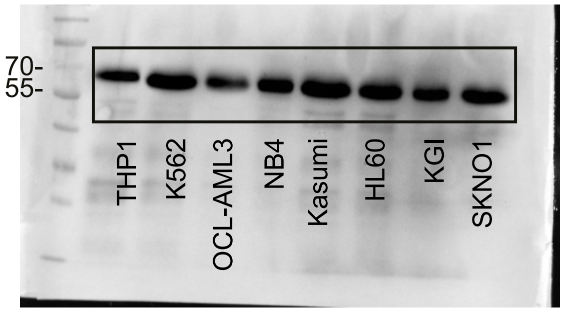

Fig. 8b. H3

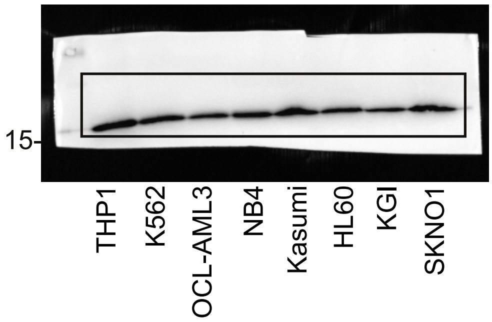

Supplementary fig. 5a.

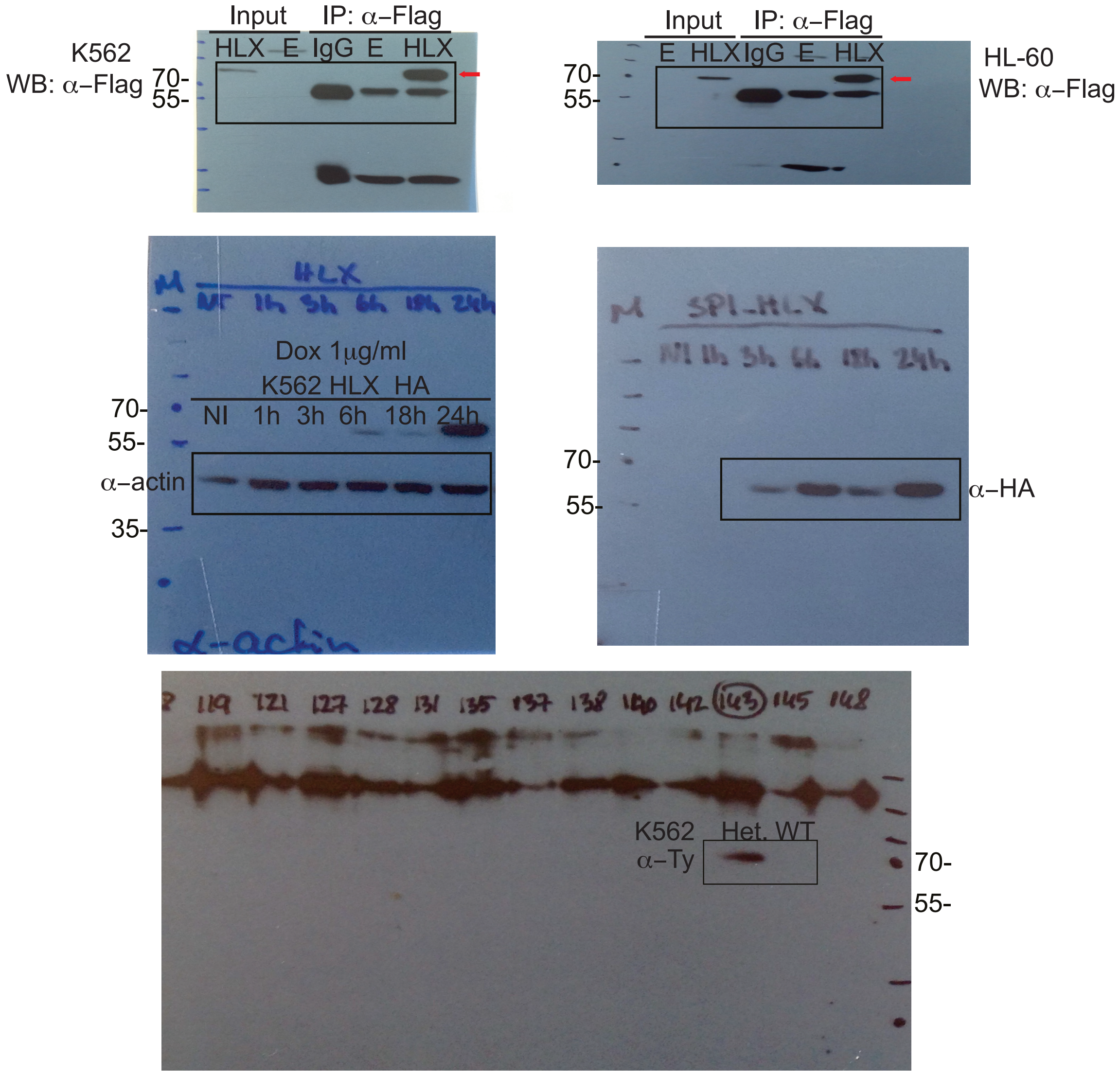

Supplementary Figure 8. Uncropped western blots

Supplementary Table 1. Cloning primers for all constructs

***HLX* cloning primers**		
Target	Forward primer	Reverse primer
Flag-Biotag HLX	TTGGCGCGCCCTTCGCAGCCGGGCTGGCTCCCTTC	CGCGGATCCCTATAAGCAGCCAAGCGCGCCTT
HLX cDNA cloning primers with attB sites	GGGGACAAGTTTGTACAAAAAAGCAGGCTTCATGTTCGCAGCCGGGCTGGCTCC	GGGGACCACTTTGTACAAGAAAGCTGGGTCCTATAAGCAGCCAAGCGCGCCTT
HLX cDNA cloning primer without stop codon	Same as above	GGGGACCACTTTGTACAAGAAAGCTGGGTCTAAGCAGCCAAGCGCGCCTTGGG
GFP cDNA cloning primers with attB sites	GGGGACAAGTTTGTACAAAAAAGCAGGCTTCATGGTGAGCAAGGGCGAGGA	GGGGACCACTTTGTACAAGAAAGCTGGGTCCTTGTACAGCTCGTCCATGC
**Human gRNA primers**		
HLX exon2	CACCGTGCGAAGAACTGGCCGGCCG	AAACCGGCCGGCCAGTTCTTCGCAC
HLX exon4	CACCGCTCAGTCCGCTCCGTGTCGC	AAACGCGACACGGAGCGGACTGAGC
for 3' UTR insertion	CACCGTACTAGGGCGGAGGGGATCC	AAACGGATCCCCTCCGCCCTAGTAC
ATP11b peak a	CACCGCGGAGCAAAGTATGGGCCA	AAACTGGCCCATACTTTGCTCCGCC
ATP11b peak b	CACCAGGCTTACAGCCCTCAGACG	AAACCGTCTGAGGGCTGTAAGCCTC
**Primers for checking gRNA indels or insertion**		
HLX exon2	CGGGTTCTCTCTTGACTTCG	TTTTTCCGTACCTGGAAACG
HLX exon4	CAAAAGGACAAGGACAAGGAG	CGCTGCTGAAGCTGAAACTA
Complete deletion exon 2-4 WT band) HLX	CGGGTTCTCTCTTGACTTCG	TTTTTCCGTACCTGGAAACG
Complete deletion exon 2-4 (KO band) HLX	Same as above	CGCTGCTGAAGCTGAAACTA
ATP11b	GGAAAGTGTCCCCTTTGCAT	CTCAGGTCTGGAAGCCTTTG
**Human primers for cloning sh-*HLX***		
HLX shRNA	CCGGCCCTATGCTGTGCTCACGACTCGAGTCGTGAGCACAGCATAGGGTTTTTG	AATTCAAAAACCCTATGCTGTGCTCACGACTCGAGTCGTGAGCACAGCATAGGG
**Sequence of 3xTy insertion**		
3xTy insert flanking HLX	TCGAGCTTGGCGGCGGCGGCGCCTCGGAGCTTCTCCCTGCAACACAGCCCACAGCCAGCAGCGCTCCCAAAAGCCCCGAGCC	CGGCTCCACGCTGCGTCTCCTGCCCTACCAAGTCGCCAACATACAGTAAGCACAAAACCCAGCGCATGGTTGGGAGGCTGCA

	AGCCCAAGGCGCGCTTGGCTGCT TAGGAGGAGAGGTGCACACCAAC CAGGACCCCCTGGACGCCGAAGT CCATACAAATC AGGATCCTCTGGATGCCGAAGTG CACACCAATCAGGATCCCCTGGA CGCTTAGACGCTTAGACTGTACTA GGGCGGAGGG GATCCGGGCCTTGCGTGCAGCCT CCCAACCATGCGCTGGGTTTTGT GCTTACTGTATGTTGGCGACTTGG TAGGGCAGGA GACGCAGCGTGGAGCCGAGCT	CGCAAGGCCCGGATCCCCTCCGCC CTAGTACAGTCTAAGCGTCTAAGCG TCCAGGGGATCCTGATTGGTGTGCA CTTCGG CATCCAGAGGATCCTGATTTGTATG GACTTCGGCGTCCAGGGGGTCCTG GTTGGTGTGCACCTCTCCTCCTAAG CAGCCA AGCGCGCCTTGGGCTGGCTCGGGG CTTTTGGGAGCGCTGCTGGCTGTG GGCTGTGTTGCAGGGAGAAGCTCC GAGGCGCC GCCGCCGCCAAGC
Primers for checking 3xTy insertion (Set I)	GGCTGCTTAGGAGGAGAGGT	ACCAAGTCGCCAACATACAG
Primers for checking 3xTy insertion (Set II)	TATTAAGGCCCCGGTCACTG	As above

Supplementary Table 2. Zebrafish and human qPCR and ChIP-qPCR primers

**Zebrafish qPCR primers**		
**Gene**	**Forward primer**	**Reverse Primer**
atp5d	TCAATGACGATGGTTCCTCTAAG	TTCTCCAGGTTCGCTTTCG
atp5a1	TTCTTGGAGCCGACACTGGA	CGAACACCACAACACCAACG
atp5h	TGGTGGATGAGTTTGAGAAGAAG	ACGAGCTTTAGATGCTTCCAG
atp6v1g1	TCGCAGAAGCCCGTAAAAG	AGCAGAGTTTCCATGAGACC
cpa5	TGGAGGCACTATTGACTGGA	GATTGGCTGGCAGAATAAAA
c-myb1	TGATGCTTCCCAACACAGAG	TTCAGAGGGAATCGTCTGCT
chuck	TCCAGAATCAAGACCGGGTG	TGAGCTCCGTGTGGTGTTTT
cox5aa	GTCACGGCCACAGTATACAG	TTCCTCAACTCCCAAGCATC
cox7c	TCGCACAGATCCAACACTTC	GCCTCCACTTGTTCTCTACTG
cox5ab	GGTCACCGGAGCTTCAGGAT	TCGAGCCGAGAGGTAGAAAAACC
cox15	ATTAGCTTACATTCTCCCCGC	CTTCACCATGTACCATCCCAG
cox17	AGCGATAGAGGGAGCAGAG	GCTCTCATGCATTCCTTGTG
cpox	TGTGAGGAGCTGCGCTAAAA	CGTGCATGTACTCCCACCTT
cyyr1	CTGTCAGGAACGGCTATCTCC	ACCCTGAGTCACTGTGTTGATG
eif2b5	CAACTCGCTCAAGTACGCCT	CAAAGACAGACTCACAGGCAG
gata1	AGCGCTCTATTCAACTGGGG	GATGAGGGGTCTGTTCTGGC
hlx1	TATCCACAGACTTCGAGCCG	AAGCGTTTCTCCAGGCCTTT
mpo	GGGGCAGAAGAAGAAAGTCC	CCCTTGCTAAACTCTCATCTCG
mfap4	TGCTCTCAGATGGGAAAGATG	GCCAGTATTCTCCCTCCACA
mpeg	GTGAAAGAGGGTTCTGTTACA	GCCGTAATCAAGTACGAGTT
ndufa4l	TGCACCATGTCTCTGTCATATC	TGTAGTCCATGTTCACAGCG
ndufb4	ACCCGCATTTCAGACCATC	AGTCCCGGCATCTATTTTGG

ndufb5	ATCCAGCCCTCATCCTTCTA	GTCTCTGCTAACTCACAATCTCC
ndufb6	CTGGGTCCTGCATTACTATCTG	ACTGTCTTCGGGAAAATCTGG
ndufs3	TCCCTTCAAAGCAGAACCG	CTCATACCAGTTAGACGCCTG
ndufb8	AACATGAGGCCTGAGGATTATC	CATCGGTTCTCCCCAGTTTC
pparaa	AGCAGGAACAGCTTGTCTACC	ATCAATCATCAGTCTCTGCTGC
pparab	GAACCGAAACAAGTGCCAATAC	CCTTCAACCTTAGCTTCTCCG
pparda	AGAAGGCCAGAAACATCCTG	GAACTCCGATCTCCTTATTGGG
ppardb	CCTCAAAACACTGGCCAAGC	GGATCACAAAAGGCGCAGTG
pparg	CTCTCCGCTGATATGGTGGAC	GTCGATGCCTGATATGCTGC
rpl34	AAACAAGACCAGGCTGTCCC	ATGGAACCACCATAGGCTCG
tal1	TGAAATCCGAGCAATTTCCGC	CCTCTCCTGGACAGCTCGAT
tgfb3	ACAATGACCTGCCCTACTGC	CTCGATTCTCTGCTCGGTCC
mito DNA	CAAACACAAGCCTCGCCTGTTTAC	CACTGACTTGATGGGGGAGACAGT
nuclear DNA	ATGGGCTGGGCGATAAAATTGG	ACATGTGCATGTCGCTCCCAAA
**Human qPCR primers**		
ATP11b	GCAGCAGCTGGGTTTTGAC	AGGCCATTCTGAGGAAACCTG
HLX	CCCTCCAGCAAAGACCTCAAA	AGGATTGCAGAAGCCTCGTT
NDUFA2	AGGGACTTCATTGAGAAACGC	TGTTCAAAGGGACATTCGTCTC
NDUFA11	ATCGAGATTGCGGGCTATG	GGATTGAGTGTGACTCTGTAGG
NDUFA6	GTGAAAATGGGACGGGATAAAG	GCATAACATGTGTCCGCTG
NDUFAB 1	ATCCAGGACCGTGTTCTTTAC	AAACCCAAATTCGTCTTCCATG
NDUFB2	ATGTGGTTCTGGATTCTCTGG	GGAAGGATCAGGATACGGAAAG
NDUFB3	ACAATGGAAGATAGAAGGGACA C	AAGAATACATCAGAAAAGGAAACACT C
NDUFB4	AGACCCACTCCTAAAAACTCAC	TGAGAGGTGAAATGTTCGATCC
NDUFB5	TTCCTCACTCGTGGCTTTC	CGCCTGTCATAGAATCTAGAAGG
NDUFC1	AGCATCCACAGCCATACATC	ATCAGGATACATTGCCTTCTCAG
NDUFC2	ATTGATAACCTAATCCGGCGG	TCCAAACATTTCACGGTCCC
NDUFS2	AGTCCGATTGCCGATTCAG	GTGGGCTGTTTCTTTGCTTG
NDUFS3	TCCCAACTCGGCAAAACC	TGCCTTGAACACAGAGACAG
PPARδ	GGGTGTTGCATCAGAGATGA	GGAAGGGGAAAAGAATTGGA
RAB7α	AAGGAGGTGATGGTGGATGA	CCACACCGAGAGACTGGAAC
tRNA-Leu(UUR) mito. DNA	CACCCAAGAACAGGGTTTGT	TGGCCATGGGTATGTTGTTA
β2-microglobulin nuc. DNA	TGCTGTCTCCATGTTTGATGTAT CT	TCTCTGCTCCCCACCTCTAAGT
**Human ChIP-qPCR primers**		
ACL1	AATTTCCAGGGACCAGTAAGAC	GCCTGGGTCTTCTCTCATTATT
ACSL4	GAACACAGACACACACAGAGAG	TCAAGGAGTTGGCAGGATTG
FOXO1	CCCACCCATATGCAAAGTCTAT	AGGCTGTATTCACCACAAAGT

KLF6	CCACGATAATGTCTGGAGTATCT T	CCTCTACTAACTCTGTATTGCTAGTT
PPARδ	GGGTGTTGCATCAGAGATGA	GGAAGGGGAAAAGAATTGGA
Negative control	TGACAGAACTGGCATGGAAA	TGTGTAAAGTTGGATGGCAAA

Supplementary Table 3. Files used in the comparison of HLX ChIP-seq with ChIP-seq from different histone marks

Name of .bam file	Description
wgEncodeBroadHistoneK562CtcfStdAlnRep1.bam	CTCF replicate 1
wgEncodeBroadHistoneK562H3k4me1StdAlnRep1.bam	H3K4me1 replicate 1
wgEncodeBroadHistoneK562H3k4me2StdAlnRep1.bam	H3K4me2 replicate 1
wgEncodeBroadHistoneK562H3k4me3StdAlnRep1.bam	H3K4me3 replicate 1
wgEncodeBroadHistoneK562H3k9acStdAlnRep1.bam	H3K9ac replicate 1
wgEncodeBroadHistoneK562H3k9me1StdAlnRep1.bam	H3K9me1 replicate 1
wgEncodeBroadHistoneK562H3k27acStdAlnRep1.bam	H3K27ac replicate 1
wgEncodeBroadHistoneK562H4k20me1StdAlnRep1.bam	H4K20me1 replicate 1

Supplementary Methods

Whole-mount *in situ* hybridization staining and analysis

Whole-mount *in situ* hybridization (WISH) was performed as previously described[1] with minor modifications. The following probes were used: *runx1*[2], *c-myb*[3], *rag1*[4], *pu.1*[5], *mpo*[6], *ephrinB2a*[7], *cpa5*[8], *mfap4*[9], *gata1*[10] and *kdrl*[11]. Digoxigenin-labeled RNA probes were synthesized by *in vitro* transcription with T7 (ThermoFischer Scientific, catalog number: EP0111) or T3 (ThermoFischer Scientific, catalog number: EP0101) RNA polymerase. The embryos were first dechorionated (when necessary) and fixed in 4% paraformaldehyde (PFA) overnight (4 ^{o}C). After fixation, the embryos were washed twice in PBS-T (0.1% v/v) and bleached in bleach solution (0.8% KOH, 9% H_2O_2, 0.1% Tween20, ddH_2O) for 10 to 45 min, depending on the embryonic stage. Embryos were washed in PBS-T and fixed in 4% PFA at RT for 2 h and dehydrated in methanol overnight. Embryos were rehydrated, permeabilised with proteinase K and refixed in 4% PFA for 20 min at RT. Embryos were washed in PBS-T and then incubated in hybridization buffer (50% formamide, 5XSSC, 0.1% Tween20, ddH_2O) at 70 ^{o}C for 30 min. Hybridization solution containing 1 ng/µL of the desired RNA probe was then added and the embryos were incubated at 70°C overnight. The embryos passed through serial washes and_blocking solution (PBS-T, 2% sheep

serum, 2 mg/mL BSA) was added for 30 min at RT. Finally, the embryos were treated overnight at 4°C in blocking solution containing 0.15U/μL anti-digoxigenin antibody (Roche, 11093274910). Finally, the embryos were washed and incubated in staining solution RT (for 10mL of staining buffer: 35μL 50mg/mL BCIP, 45μl 50mg/mL NBT) till stained. All WISH experiments were performed at least three independent times. Embryos were imaged in 100% glycerol, using a Leica stereomicroscope. Qualitative scoring (number of embryos with altered HSCs per number scored) of WISH staining was conducted manually by visual observation, blindly. The numbers at each picture represent the number of embryos that simulate the embryo in the figure relative to the total number of counted embryos. Quantitative scoring was conducted after taking pictures of stained embryos and analyzing them with Fiji software through the Color Segmentation plugin. Shortly, cells were counted in the tail region using cell counter plugin (for *runx1*) or the intensity of stained region was measured (for *rag1*). Fold change between the control and experimental group represents the average of three independent experiments.

Constructs and generation of transgenic zebrafish lines

The transgenic lines *Tg(kdrl:eGFP)*[12], *Tg(fli:Gal4) or officially Tg(fli1a:Gal4FF)ubs3*[13], *Tg(Runx:mCherry) or officially Tg(Mmu.Runx1:NLS-mCherry)*[14], *Tg(fli1a:eGFP)*[15] are described elsewhere. *Tg(Runx:Gal4)* or officially *Tg(Mmu.Runx1:GAL4)* was created by Marina Mione (requests should be addressed to the Mione lab) and contains the Gal4 gene under the control of the regulatory elements used in *Tg(Runx:mCherry)*[14], namely the mouse +23 Runx enhancer element and a mini β-globin promoter.

Human *HLX* cDNA was amplified by PCR from a human cDNA clone (Origene, NM_021958) and cloned into middle donor vector pDONR221 (Tol2kit:218), using BP clonase II (Thermo Fisher Scientific) generating pDONR221-*HLX*. The pTol2-5x*UAS:HLX-cmlc2:eGFP* construct was generated by recombining pDONR221-*HLX* into the pTol2-5x*UAS:cmlc2:eGFP* plasmid using LR clonase II (Thermo Fisher Scientific).

The *Tg(UAS:HLX-GFP)* line was generated by co-injecting 12 pg of Tol2 transposase mRNA and 25 pg of pTol2-5x*UAS:HLX-cmlc2:eGFP* construct into wild type *Tü* at the one-cell stage. Capped messenger RNA encoding Tol2 transposase was generated using pCS2FA-transposase (Tol2kit: 396) as a template, linearized with NotI, followed by *in vitro* transcription using the mMessage mMachine SP6 kit (Ambion). Individual F0 founders were outcrossed to *Tü*, and their F1 progeny were screened for the *cmlc2-EGFP* transgenesis marker. *fli*:h*HLX* OE and *Runx*:h*HLX* OE embryos were generated by crossing *Tg(fli:Gal4)* or *Tg(Runx:Gal4)* respectively with *Tg(UAS:HLX-GFP)* lines.

fli:h*HLX* and *Runx*:h*HLX* constructs used for the rescue experiments were generated by recombining pDONR221-*HLX* as middle donor vector with p5E-fli1ep (478 p5Efli1ep was a gift from Nathan Lawson (Addgene plasmid # 31160)) or p5E-*Runx*+23 (Tol2 kit:161 a gift from Owen Tamplin) as 5'entry vectors and polyA (Tol2 kit: 302) into the destination vector pDestTol2pA2 (Tol2 kit:394), using LR clonase II[16].

Cell line generation and constructs

All constructs used are described in Supplementary Methods. For **stable *HLX* overexpression** two systems were used: 1) non inducible *HLX* expression: DNA encoding full-length HLX was cloned into pEF1α-FLAG-Biotag generating pEF1α-HLX-FLAG-Biotag[17]. $4x10^6$ K562 or HL60 cells were electroporated (200 V, 950 µF, Bio-Rad) with 20 µg of pEF1α-*HLX*-FLAG-Biotag plasmid and stable cell lines were selected and maintained in puromycin (2 µg/mL). These clones were used for the ChIP-seq experiments. 2) Inducible *HLX* expression: The pDONR221-*GFP* construct was generated by recombining PCR amplified *GFP* into the pDONR221 plasmid using BP clonase II. pDONR221-*GFP* or pDONR221-*HLX* were recombined with the lentiviral gateway vector pInducer21(ORF-EG) (gift from Stephen Elledge & Thomas Westbrook (Addgene plasmid # 46948))[18], using LR clonase II (Thermo Fisher Scientific) and generating pInducer21-*GFP*-HA (*GFP*-HA) and pInducer21-*HLX*-HA (*HLX*-HA) (Primers on Supplementary Table 1). K562, HL60 and THP-1 cells were transduced with lentivirus using spinoculation (8

µg/mL polybrene, 2000xg, 90 min) with pInducer21-*GFP*-HA or pInducer21-*HLX*-HA to generate inducible GFP-HA and *HLX*-HA overexpressing cell lines. Cells were sorted for GFP. The K562 and HL60 HLX overexpressing cell lines were used for an independent ChIP-qPCR verification and qPCR experiments after induction of HLX expression with 1mg/mL doxycycline. The THP1 HLX overexpressing cell lines and the respective controls were used for all the experiments involving THP1 cells.

For the generation of **K562 *HLX*-KO cell lines** two gRNAs on exon 2 and 4 were cloned in pSpCas9(BB)-2A-GFP (PX458). 20 µg of these constructs were used in electroporation of K562 cells (200 V, 950 µF, Bio-Rad) and single fluorescent cells were FACS-sorted into 96-well plates 48 h afterwards. DNA was isolated by alkaline lysis and PCR for the deletion fragment was performed (Primers on Supplementary Table 1).

To generate K562 cells lacking the HLX bound regions on *ATP11b* gene two gRNAs were designed using the CHOPCHOP tool [19] or the Crispr design tool from the Zhang lab[20] around the HLX bound region close to *ATP11b* gene The gRNAs were cloned in pSpCas9(BB)-2A-GFP (PX458) (a gift from Feng Zhang (Addgene plasmid # 48138)[21]) and verified by sequencing according to the instructions by Cong et al[20]. gRNAs were electroporated (200 V, 950 µF, Bio-Rad) in K562 cells transiently and cells were checked for deletion by PCR. (gRNA sequences and primers to check the deletion created are provided in Supplementary Table 1).

To generate the **3xTy *HLX*** the following plasmids were constructed. gRNA targeting the 3'end of the HLX locus was cloned in pSpCas9(BB)-2A-GFP (PX458)[20] (oligo sequences on Supplementary Table 1). The linear oligo used as template to insert the 3xTy is described in Supplementary Table 1. The gRNA together with the oligo were electroporated (200 V, 950 µF, Bio-Rad) in K562 cells. Clones were screened using PCR (primers in Supplementary Table 1), followed by sequencing and Western blot.

RNA-seq libraries

For zebrafish RNA-seq experiements, sorted *kdrl:*GFP cells from control, *hlx1* MO or *fli:*mCherry cells from h*HLX* OE fish at 48 hpf were used. RNA was extracted using the RNeasy Plus Micro kit (Qiagen). The SMART-Seq v4 Ultra Low Input RNA kit (Clontech Laboratories) was used for cDNA synthesis. The sequencing libraries were prepared using the NEBNext® Ultra™RNA Library Prep kit (NEB). For the RNA-seq experiments in K562 and CD34+ cells RNA was extracted using RNA Clean & Concentrator-% (Zymo Research). For library preparation the NEBNext® Ultra™RNA Library Prep (total RNA) kit (NEB) was used. Library preparation and sequencing was performed by the Max Planck Institute of Immunobiology and Epigenetics Sequencing Facility.

RNA-seq preparation and analysis methodology

Paired-end 75 bp reads for control and experimental samples were generated with NextSeq500 or Illumina 2500. RNA-seq raw sequencing data from zebrafish (control, *hlx1* morphant and *HLX* OE) were trimmed to 35 bp and aligned to zebrafish genome version GRCz10/danRer10, while RNA-seq data from K562 *HLX*-KO and CD34$^+$ cells were aligned to human genome version GCCh37/hg19 with the tophat algorithm (version 2.0.9)[22] and the use of «--b2-very-sensitive» parameter. Samtools (version 0.1.19)[23] were used for data filtering and file format conversion while the HT-seq count (version 0.6.1p1) algorithm[24] was applied to assign aligned reads to exons using the following command line «htseq-count –s no –m intersection -nonempty». Differentially expressed genes were identified with the use of the DESeq R package[25], and genes with fold change cut-off 2 and P<=0.05 were considered to be differentially expressed (DEGs) in K562 cells or zebrafish cells, while all genes with fold change cut-off 1.5 were considered in CD34$^+$ cells without taking into account p-values. Heatmaps that were constructed to depict DEGs or selected genes were generated with R/Bioconductor. A summary of the differentially expressed genes can be found in Supplementary Data 1 and 5.

ATAC-seq libraries

Sorted kdrl:GFP+ zebrafish cells from *hlx1*MO and control were prepared for ATAC-seq according to a protocol described before[26,27]. Briefly 50,000 cells were spinned down at 500 ×g for 5 min, 4°C. Cells were washed with 50 µL of cold 1x PBS buffer and spinned down at 500 ×g for 5 min, 4°C. Cells were resuspended in 50µL of cold lysis buffer (10 mM Tris-HCl, pH 7.4, 10 mM NaCl, 3 mM $MgCl_2$, 0.1% IGEPAL CA-630) and spinned down immediately at 500 ×g for 10 min, 4°C. Cells were resuspended in transposition reaction mix (25µL TD buffer, 2.5µL TDE1 transposase, 22.5µL nuclease free H_2O, Illumina Nextera DNA Sample preparation kit FC-121-1030) and incubated at 37°C for 30 min. Transposased DNA was purified using a Qiagen MinElute PCR Purification Kit (28004). DNA fragments were PCR amplified with 2.5µL of Nextera PCR primers (Illumina) with NEBNext High-Fidelity 2x PCR Master Mix (NEB, M0541) for 1 cycle of 72°C for 5 min, 98°C for 30 sec, followed by 5 cycles of 98°C for 10 sec, 63°C for 30 sec, 72°C for 1 min. qPCR was used to determine the extra cycled needed for each sample and the PCR was continued for the required amount of cycles. DNA was purified using a Qiagen MinElute PCR Purification Kit (28004). Sequencing was performed at the Max Planck Institute of Immunobiology and Epigenetics Sequencing Facility.

ATAC-seq analysis methodology

ATAC-seq paired-end 75 bp reads for control and *hlx1*MO zebrafish endothelial cells were generated with NextSeq500. Bowtie2 algorithm[28] (version 2.1.0) and «--very-sensitive» parameter were used for aligning ATAC-seq data to the zebrafish genome version GRCz10/danRer10. Samtools (version 0.1.19)[23] were used for data filtering and file format conversion. Duplicate reads were removed before peak calling. The MACS (version 1.4.2) algorithm[29] was used for ATAC-seq peak identification with default p-value 1-E05. All bam files were converted to bedgraphs with genomeCoverageBed and MACS2 (version 2.1.0) bdgdiff command was used in order to identify differential enrichment in the accessible regions between control and *hlx* MO samples, with default options. Gene annotation (25 kB away from the gene) and genomic distribution of accessible regions identified

by MACS was performed with bedtools[30] and graph representation (heatmaps) of the tag – read density around TSS was performed with seqMiner (version 1.3.3) software[31]. High resolution tracks of ATAC coverage were calculated as the number of fragment ends mapping to each genomic position, after adjusting the 5' and 3' coordinates by +4 and -5 bp, respectively, to account for the offsets of transposase cut sites[26], and considering only sub-nucleosomal (<150 bp) fragments for analysis. A summary of ATAC-seq peaks calculated by using all the accessible regions or the sub-nucleosomal fragments can be found in Supplementary Data 2.

Chromatin Immunoprecipitation

For ChIP-seq experiments, FLAG M2 magnetic beads were used (Sigma-Aldrich) for K562 and HL60 cells and HA matrix beads (Sigma Aldrich/Roche) for THP1 cells. For ChIP-qPCR experiments, FLAG M2 antibody (Sigma-Aldrich, F1804), HA antibody (rat α-HA Sigma, 000000011867423001), Ty1 antibody (MAb-054-050, Diagenode), rabbit IgG (Millipore, 12-370), mouse IgG (Millipore, 12-371) rat IgG (Millipore, NIO4) were used.
ChIP experiments were performed as described previously[32,33]. Briefly, 10^8 cells for K562, HL60 or THP1 cell lines were crosslinked by the addition of 1/10 volume 11% fresh formaldehyde for 10 min at room temperature. The crosslinking was quenched by the addition of 1/20v/v 2.5M Glycine. Cells were washed twice with ice-cold PBS and the pellet was flash-frozen in liquid nitrogen. Cells were kept at -80°C until the experiments were performed. Cells were lysed in 10 mL of Lysis buffer 1 (50mM HEPES-KOH, pH 7.5, 140mM NaCl, 1mM EDTA, 10% glycerol, 0.5% NP-40, 0.25% Triton X-100, protease inhibitors) for 10min at 4°C. After centrifugation, cells were resuspended in 10 mL of Lysis buffer 2 (10mM Tris HCl, pH 8.0, 200mM NaCl, 1mM EDTA, 0.5mM EGTA, protease inhibitors) for 10 min at room temperature. Cells were pelleted and resuspended in 3 mL of Sonication buffer per 10^8 cells (10mM Tris-HCl, pH 8.0, 100mM NaCl, 1mM EDTA, 0.5mM EGTA, 0.1% Na-Deoxycholate, 0.05% N-lauroylsarcosine, protease Inhibitors) and sonicated in Bioruptor sonicator for 40 cycles of 30s of sonication followed by 1min resting intervals. Samples were centrifuged for 10 min at 18,000 g and the

pellet was discarded. Approximately $3x10^7$ cells were used for each immunoprecipitation. Prior to the immunoprecipitation, 50µL of FLAG or HA matrix beads for each reaction were washed twice with PBS, 0.5% BSA twice. Cell lysates were added to the beads that were incubated at 4^oC overnight. Beads were washed once with wash buffer I (20mM Tris-HCl (pH 8), 150mM NaCl, 2mMEDTA, 0.1% SDS, 1%Triton X-100), once with wash buffer II (20mM Tris-HCl (pH 8), 500 mM NaCl, 2 mM EDTA, 0.1% SDS, 1%Triton X-100), once with wash buffer III (10 mM Tris-HCl (pH 8), 250 nM LiCl, 2 mM EDTA, 1% NP40), once with TE and finally resuspended in 200µL elution buffer (50 mM Tris-HCl, pH 8.0, 10 mM EDTA and 0.5%–1% SDS) by heating at 65^oC for 30 min in a shaking heat block. 50µL of cell lysates prior to addition to the beads were kept as input. Crosslinking was reversed by incubating samples at 65^oC for at least 6 hr. After the reversal of crosslinking cells were treated with RNase and proteinase K and the DNA was extracted by Phenol/Chloroform extraction.

For ChIP-qPCR another sonication strategy was followed[34]. Briefly, crosslinked cells were resuspended in Farnham buffer (5 mM PIPES pH 8.0, 85 mM KCl, 0.5% Igepal and 1x protease inhibitors). 10^7 cells were transferred into 1 mL Covaris sonication tubes and sonicated (peak power 75, duty factor 2, cycles/burst 200) until 80-90% of purified nuclei were observed. Nuclei were washed two times with Farnham buffer and resuspended in shearing buffer (10 mM Tris-HCl pH 8, 1 mM EDTA, 0.1% (v/v) SDS, 1x protease inhibitors) and sonicated again (peak power 140, duty factor 5, time 25-30 min). Immunoprecipitation was further performed as described above. ChIP-qPCR primers can be found in Supplementary Table 2.

ChIP-seq libraries

Libraries for ChIP samples were prepared using NEBNext® Ultra™ II DNA Library Prep Kit (Illumina) by the Sequencing Facility of Max Planck Institute of Immunobiology and Epigenetics.

ChIP-seq analysis

A summary of ChIP-Seq peaks can be found in Supplementary Data 4.

ChIP-seq single-end 50 bp reads for HLX in K562 (two replicates), HL-60 (one replicate) cells and their corresponding inputs were generated with Illumina HiSeq2500. ChIP-seq paired-end 75 bp reads for HLX (two replicates), H3K27ac (two replicates) and their corresponding input in THP1 cells were generated with NextSeq 500. Bowtie2 algorithm[28] (version 2.1.0) and «--very-sensitive» parameter were used for aligning ChIP-seq data to the human genome version GCCh37/hg19. Samtools (version 0.1.19)[23] were used for data filtering and file format conversion. Duplicate reads were removed before peak calling. The MACS (version 1.4.2) algorithm[29] with default p-value 1-E05 was used for ChIP-seq peak identification in K562 and HL60 cell samples (single end) and MACS (version 2.1 .0) with default q-value 0.05 for THP1 cells (paired-end). All bam files were converted to bedgraphs with genomeCoverageBed. Gene annotation and genomic distribution of the peaks identified by MACS was performed with bedtools[30] and graph representation (heatmaps) of the tag – read density around TSS was performed with SeqMiner (version 1.3.3) software[31].

All *.bam files that were used for Fig. 4f were downloaded from the ENCODE database (http://hgdownload.soe.ucsc.edu/goldenPath/hg19/encodeDCC/wgEncodeBroadHistone/). For the heatmap the first replicate from each experiment for H3K4me1/me2/me3, H3K9me1/ac, H3K27ac, H4K20me1 and CTCF was used. The files that were used as shown in ENCODE database are presented in the Supplementary Table 3. Data used in Fig. 5c were downloaded from previously published ATAC-seq data[35]. The following samples were used: a) hematopoietic stem cell samples (two replicates, GSM1937416 & GSM1937417). b) acute myeloid leukemia, pre-leukemic hematopoietic stem cell samples (one replicate, GSM1937494), c) acute myeloid leukemia, leukemia stem cell sample (one replicate, GSM1937494). Heatmaps were generated using SeqMiner as described above.

Gene ontology, pathway and network analysis

Gene ontology and pathway analysis of the differentially expressed genes from RNA-seq and peaks from ChIPseq (+/- 5kb from TSS) and ATACseq (+/- 25 kB from TSS) was performed with DAVID knowledgebase[36,37] and Ingenuity Pathway Analysis software (IPA, Ingenuity® Systems, www.ingenuity.com) with the default settings. Only pathways and biological processes with p-value <=0.05 were considered to be significantly enriched. The enriched regions from each ChIP-seq in human samples[38] and ATAC-seq in zebrafish samples[39] were imported into Genomic Regions Enrichment of Annotations Tool (GREAT). The analysis was done according to the default settings. We considered categories with p-values < 10^4 as significant.

Motif analysis

Motif analysis for HLX ChIP-seq and ATAC-seq peaks was performed with MEME-ChIP[40] and “findMotifsGenome.pl” for masked motifs from HOMER software[41].

Digital genomic footprinting for ATAC-Seq

To attain sufficient sequencing depth to perform digital genomic footprinting (DGF) on ATAC-Seq data produced in this work, aligned bam files were merged using samtools merge (version 1.3.1)[23] following sorting via samtools merge and subsequently indexed using samtools index. This step yielded 176,081,387 and 192,905,736 single-end reads for HLX WT and morphant data sets, respectively, both numbers being greater than the minimum 100 million reads required to perform DGF in mammalian cells[42]. DGF was performed using dnase_footprints of the Wellington pyDNase package[43] (version 0.2.4) on total merged ATAC peaks using -A as a parameter to enable ATAC mode, resulting in coordinate shift 5’ and 3’ by +4 and -5 bp, respectively. Motif overrepresentation and co-occurrence enrichment analyses were performed as previously described[44] on HLX WT-only footprints versus all HLX morphant footprints and vice-versa. Briefly, motif bootstrapping was performed in WT-only and MO-only footprints using HOMER motifs corresponding to de novo found motifs found in WT- and MO-

specific footprints. Co-occurrences were derived using pyBedTools intersection_matrix from bed files corresponding to footprinted motifs. To compute background co-occurrences, 1000 random iterations were performed in equally sized samplings (19438 and 15281, respectively) from global MO and WT footprints, respectively. Mean and standard deviation of background co-occurrences (μ and σ) were subsequently used for co-occurrence enrichment computation expressed as Z=(x-μ/σ), where x is the observed co-occurrence for each footprinted motif. Hierarchical clustering of Z-scores was then performed using complete linkage, spearman correlation. Additionally, we performed t-tests based on the footprinting occupancy scores at WT-specific Hoxc9 motifs using the WT and MO ATAC data, and the same at MO-specific AP-1 motifs. Footprinting occupancy scores were retrieved using a custom script of the pyDNase package, dnase_fos_scorer.py[45], using the -A switch for ATAC data. Footprinting occupancy scores (FOS) between two experiments are directly comparable as they are computed using the following formula: **FOS = (C+1)/L + (C+1)/R.** Whereby C is the average number of reads over the candidate region and L,R the average number of reads left, right of the candidate region, respectively[46]. FOSs were first tested for normality to make sure the t-test can apply using a Shapiro-Wilks test in R. As the resulting p-values indicated that FOSs did not follow a normal distribution, we performed a log2 transform, which resulted in normal data; following are the p-values for the Shapiro-Wilks normality test (p>=0.05 implies the data is normal)

Hoxc9 WT-specific, log2 WT footprinting occupancy scores, **p= 0.5599** (was 6.077e-05 without log2 transform)

Hoxc9 WT-specific, log2 MO footprinting occupancy scores, **p=0.38** (was 3.639e-06 without log2 transform)

AP-1 MO-specific, log2 WT footprinting occupancy scores, **p= 0.3597** (was 4.695e-10 without log2 transform)

AP-1 MO-specific, log2 MO footprinting occupancy scores, **p= 0.1146** (was 8.907e-11 without log2 transform)

to the same chromosomal region, and not assuming any direction in the relationship between both samples. Following are the t-test p-values between FOSs at Hoxc9 and AP-1 motifs between WT and HLX-MO ATAC datasets:

Hoxc9 WT-specific, log2 WT vs MO footprinting occupancy scores, **p= 2.9884E-34** (was 8.64471E-31 without log2 transform)
AP-1 MO-specific, log2 WT vs MO footprinting occupancy scores, **p= 1.24262E-37** (was 7.34244E-35 without log2 transform)

Network construction

Cytoscape[47] and Metascape[48] were used for network construction.

Hierarchical differentiation tree

The Hierarchical differentiation trees for HLX expression in mouse and human were performed using BloodSpot[49].

Supplementary References:

1 Thisse, C. & Thisse, B. High-resolution in situ hybridization to whole-mount zebrafish embryos. *Nat Protoc* **3**, 59-69, doi:10.1038/nprot.2007.514 (2008).
2 Burns, C. E. *et al.* Isolation and characterization of runxa and runxb, zebrafish members of the runt family of transcriptional regulators. *Exp Hematol* **30**, 1381-1389 (2002).
3 Bolli, N. *et al.* cpsf1 is required for definitive HSC survival in zebrafish. *Blood* **117**, 3996-4007, doi:10.1182/blood-2010-08-304030 (2011).
4 Schorpp, M. *et al.* Conserved functions of Ikaros in vertebrate lymphocyte development: genetic evidence for distinct larval and adult phases of T cell development and two lineages of B cells in zebrafish. *J Immunol* **177**, 2463-2476 (2006).
5 Lieschke, G. J. *et al.* Zebrafish SPI-1 (PU.1) marks a site of myeloid development independent of primitive erythropoiesis: implications for axial patterning. *Dev Biol* **246**, 274-295, doi:10.1006/dbio.2002.0657 (2002).
6 Lieschke, G. J., Oates, A. C., Crowhurst, M. O., Ward, A. C. & Layton, J. E. Morphologic and functional characterization of granulocytes and macrophages in embryonic and adult zebrafish. *Blood* **98**, 3087-3096 (2001).
7 Lawson, N. D. *et al.* Notch signaling is required for arterial-venous differentiation during embryonic vascular development. *Development* **128**, 3675-3683 (2001).
8 Dobson, J. T. *et al.* Carboxypeptidase A5 identifies a novel mast cell lineage in the zebrafish providing new insight into mast cell fate determination. *Blood* **112**, 2969-2972, doi:10.1182/blood-2008-03-145011 (2008).
9 Walton, E. M., Cronan, M. R., Beerman, R. W. & Tobin, D. M. The Macrophage-Specific Promoter mfap4 Allows Live, Long-Term Analysis of

Macrophage Behavior during Mycobacterial Infection in Zebrafish. *PLoS One* **10**, e0138949, doi:10.1371/journal.pone.0138949 (2015).

10 Lyons, S. E. *et al.* A nonsense mutation in zebrafish gata1 causes the bloodless phenotype in vlad tepes. *Proc Natl Acad Sci U S A* **99**, 5454-5459, doi:10.1073/pnas.082695299 (2002).

11 Thompson, M. A. *et al.* The cloche and spadetail genes differentially affect hematopoiesis and vasculogenesis. *Dev Biol* **197**, 248-269, doi:10.1006/dbio.1998.8887 (1998).

12 Jin, S. W., Beis, D., Mitchell, T., Chen, J. N. & Stainier, D. Y. Cellular and molecular analyses of vascular tube and lumen formation in zebrafish. *Development* **132**, 5199-5209, doi:10.1242/dev.02087 (2005).

13 Herwig, L. *et al.* Distinct cellular mechanisms of blood vessel fusion in the zebrafish embryo. *Curr Biol* **21**, 1942-1948, doi:10.1016/j.cub.2011.10.016 (2011).

14 Tamplin, O. J. *et al.* Hematopoietic stem cell arrival triggers dynamic remodeling of the perivascular niche. *Cell* **160**, 241-252, doi:10.1016/j.cell.2014.12.032 (2015).

15 Lawson, N. D. & Weinstein, B. M. In vivo imaging of embryonic vascular development using transgenic zebrafish. *Dev Biol* **248**, 307-318 (2002).

16 Kwan, K. M. *et al.* The Tol2kit: a multisite gateway-based construction kit for Tol2 transposon transgenesis constructs. *Dev Dyn* **236**, 3088-3099, doi:10.1002/dvdy.21343 (2007).

17 Kim, J., Cantor, A. B., Orkin, S. H. & Wang, J. Use of in vivo biotinylation to study protein-protein and protein-DNA interactions in mouse embryonic stem cells. *Nat Protoc* **4**, 506-517, doi:10.1038/nprot.2009.23 (2009).

18 Meerbrey, K. L. *et al.* The pINDUCER lentiviral toolkit for inducible RNA interference in vitro and in vivo. *Proc Natl Acad Sci U S A* **108**, 3665-3670, doi:10.1073/pnas.1019736108 (2011).

19 Montague, T. G., Cruz, J. M., Gagnon, J. A., Church, G. M. & Valen, E. CHOPCHOP: a CRISPR/Cas9 and TALEN web tool for genome editing. *Nucleic Acids Res* **42**, W401-407, doi:10.1093/nar/gku410 (2014).

20 Cong, L. *et al.* Multiplex genome engineering using CRISPR/Cas systems. *Science* **339**, 819-823, doi:10.1126/science.1231143 (2013).

21 Ran, F. A. *et al.* Genome engineering using the CRISPR-Cas9 system. *Nat Protoc* **8**, 2281-2308, doi:10.1038/nprot.2013.143 (2013).

22 Kim, D. *et al.* TopHat2: accurate alignment of transcriptomes in the presence of insertions, deletions and gene fusions. *Genome Biol* **14**, R36, doi:10.1186/gb-2013-14-4-r36 (2013).

23 Li, H. *et al.* The Sequence Alignment/Map format and SAMtools. *Bioinformatics* **25**, 2078-2079, doi:10.1093/bioinformatics/btp352 (2009).

24 Anders, S., Pyl, P. T. & Huber, W. HTSeq--a Python framework to work with high-throughput sequencing data. *Bioinformatics* **31**, 166-169, doi:10.1093/bioinformatics/btu638 (2015).

25 Anders, S. & Huber, W. Differential expression analysis for sequence count data. *Genome Biol* **11**, R106, doi:10.1186/gb-2010-11-10-r106 (2010).

26 Buenrostro, J. D., Giresi, P. G., Zaba, L. C., Chang, H. Y. & Greenleaf, W. J. Transposition of native chromatin for fast and sensitive epigenomic

profiling of open chromatin, DNA-binding proteins and nucleosome position. *Nat Methods* **10**, 1213-1218, doi:10.1038/nmeth.2688 (2013).
27 Buenrostro, J. D., Wu, B., Chang, H. Y. & Greenleaf, W. J. ATAC-seq: A Method for Assaying Chromatin Accessibility Genome-Wide. *Curr Protoc Mol Biol* **109**, 21 29 21-29, doi:10.1002/0471142727.mb2129s109 (2015).
28 Langmead, B. & Salzberg, S. L. Fast gapped-read alignment with Bowtie 2. *Nat Methods* **9**, 357-359, doi:10.1038/nmeth.1923 (2012).
29 Zhang, Y. *et al.* Model-based analysis of ChIP-Seq (MACS). *Genome Biol* **9**, R137, doi:10.1186/gb-2008-9-9-r137 (2008).
30 Quinlan, A. R. & Hall, I. M. BEDTools: a flexible suite of utilities for comparing genomic features. *Bioinformatics* **26**, 841-842, doi:10.1093/bioinformatics/btq033 (2010).
31 Ye, T. *et al.* seqMINER: an integrated ChIP-seq data interpretation platform. *Nucleic Acids Res* **39**, e35, doi:10.1093/nar/gkq1287 (2011).
32 Lee, T. I., Johnstone, S. E. & Young, R. A. Chromatin immunoprecipitation and microarray-based analysis of protein location. *Nat Protoc* **1**, 729-748, doi:10.1038/nprot.2006.98 (2006).
33 Trompouki, E., Bowman, T. V., Dibiase, A., Zhou, Y. & Zon, L. I. Chromatin immunoprecipitation in adult zebrafish red cells. *Methods Cell Biol* **104**, 341-352, doi:10.1016/B978-0-12-374814-0.00019-7 (2011).
34 Arrigoni, L. *et al.* Standardizing chromatin research: a simple and universal method for ChIP-seq. *Nucleic Acids Res* **44**, e67, doi:10.1093/nar/gkv1495 (2016).
35 Corces, M. R. *et al.* Lineage-specific and single-cell chromatin accessibility charts human hematopoiesis and leukemia evolution. *Nat Genet* **48**, 1193-1203, doi:10.1038/ng.3646 (2016).
36 Huang da, W., Sherman, B. T. & Lempicki, R. A. Systematic and integrative analysis of large gene lists using DAVID bioinformatics resources. *Nat Protoc* **4**, 44-57, doi:10.1038/nprot.2008.211 (2009).
37 Huang da, W., Sherman, B. T. & Lempicki, R. A. Bioinformatics enrichment tools: paths toward the comprehensive functional analysis of large gene lists. *Nucleic Acids Res* **37**, 1-13, doi:10.1093/nar/gkn923 (2009).
38 McLean, C. Y. *et al.* GREAT improves functional interpretation of cis-regulatory regions. *Nat Biotechnol* **28**, 495-501, doi:10.1038/nbt.1630 (2010).
39 Hiller, M. *et al.* Computational methods to detect conserved non-genic elements in phylogenetically isolated genomes: application to zebrafish. *Nucleic Acids Res* **41**, e151, doi:10.1093/nar/gkt557 (2013).
40 Machanick, P. & Bailey, T. L. MEME-ChIP: motif analysis of large DNA datasets. *Bioinformatics* **27**, 1696-1697, doi:10.1093/bioinformatics/btr189 (2011).
41 Heinz, S. *et al.* Simple combinations of lineage-determining transcription factors prime cis-regulatory elements required for macrophage and B cell identities. *Mol Cell* **38**, 576-589, doi:10.1016/j.molcel.2010.05.004 (2010).
42 Boyle, A. P. *et al.* High-resolution genome-wide in vivo footprinting of diverse transcription factors in human cells. *Genome Res* **21**, 456-464, doi:10.1101/gr.112656.110 (2011).

43 Piper, J. *et al.* Wellington: a novel method for the accurate identification of digital genomic footprints from DNase-seq data. *Nucleic Acids Res* **41**, e201, doi:10.1093/nar/gkt850 (2013).
44 Obier, N. *et al.* Cooperative binding of AP-1 and TEAD4 modulates the balance between vascular smooth muscle and hemogenic cell fate. *Development* **143**, 4324-4340, doi:10.1242/dev.139857 (2016).
45 Bevington, S. L. *et al.* Inducible chromatin priming is associated with the establishment of immunological memory in T cells. *EMBO J* **35**, 515-535, doi:10.15252/embj.201592534 (2016).
46 Neph, S. *et al.* An expansive human regulatory lexicon encoded in transcription factor footprints. *Nature* **489**, 83-90, doi:10.1038/nature11212 (2012).
47 Shannon, P. *et al.* Cytoscape: a software environment for integrated models of biomolecular interaction networks. *Genome Res* **13**, 2498-2504, doi:10.1101/gr.1239303 (2003).
48 Tripathi, S. *et al.* Meta- and Orthogonal Integration of Influenza "OMICs" Data Defines a Role for UBR4 in Virus Budding. *Cell Host Microbe* **18**, 723-735, doi:10.1016/j.chom.2015.11.002 (2015).
49 Bagger, F. O. *et al.* BloodSpot: a database of gene expression profiles and transcriptional programs for healthy and malignant haematopoiesis. *Nucleic Acids Res* **44**, D917-924, doi:10.1093/nar/gkv1101 (2016).
