# Supplementary material for: A metabolic interplay coordinated by HLX regulates myeloid differentiation and AML through partly overlapping pathways
Source: Nat Commun. 2018 Aug 6;9:3090. doi: 10.1038/s41467-018-05311-4 (PMC6078963; doi:10.1038/s41467-018-05311-4)
Supplement: Supplementary file 3 — Description of Additional Supplementary Files [file 41467_2018_5311_MOESM3_ESM.pdf]

Description of Additional Supplementary Files

File Name: Supplementary Data 1
Description: RNA-seq zebrafish. Sheet 1-2. Total RNA-seq results (1) and differentially expressed genes (2) in endothelial cells of control and hlx1 MO. Sheet 3. GO analysis of hlx1 MO deregulated genes. Sheet 4. IPA analysis (Canonical Pathways) of hlx1 MO deregulated genes. Sheets 5-6. Total RNA-seq results (5) and differentially expressed genes (6) in endothelial cells of control and hHLX OE. Sheet 7. GO analysis of hHLX OE deregulated genes. Sheet 8. IPA analysis (Canonical Pathways) of hHLX OE deregulated genes

File Name: Supplementary Data 2
Description: ATAC-seq zebrafish. Sheet 1. Total ATAC-seq results. Peaks located around 25kB of a gene were assigned to respective genes. Sheet 2. Genes with differential ATAC-seq peaks in control or hlx1 MO. Sheets 3-4. GO (3) and IPA (4) analysis of differential ATAC-seq peaks. Sheet 5-6. Motif analysis in ATAC-seq peaks gained or lost in control (5) or hlx1 MO (6). Sheets 7-8. Comparison of genes with differential ATAC-seq peaks with upregulated (7) or downregulated (8) genes from hlx1 MO RNA-seq analysis. Sheet 9. IPA analysis of genes from Sheets 7,8. Sheets 10-11. Comparison of genes with differential ATAC-seq peaks with upregulated (10) or downregulated (11) genes from hHLX OE RNA-seq analysis. Sheet 12. IPA analysis of genes 2 from Sheets 10,11. Sheet 13. Sub-nucleosomal analysis of ATAC-seq and comparison to RNAseq. Sheet 14-15. GREAT GO analysis on WT (14) and hlx1 MO (15) ATAC-seq peaks.

File Name: Supplementary Data 3
Description: TCGA patients samples analysis. Sheet 1. Genes from RNA-seq of AML patients whose expression correlates or anticorrelates with HLX expression (Pearson Correlation Coefficient above 0.4, expression cut-off of 50 RSEM). Sheet 2-3-4. GO analysis of HLX correlated genes (2), anti-correlated genes (3) and IPA of both correlated and anti-correlated genes (4).

File Name: Supplementary Data 4
Description: ChIP-seq. Sheet 1. Enriched regions for HLX in K562 cells. Sheet 2-3. GO analysis (2) and IPA (3) in HLX bound genes in K562 cells. Sheet 4-5. Motif analysis in K562 cells before (4) or after masking of known transcription factors (5). Sheet 6. Enriched regions for HLX in HL60 cells. Sheet 7-8. GO analysis (7) and IPA (8) in HLX bound genes in HL60 cells. Sheet 9-10. Motif analysis in HL60 cells before (9) or after masking of known transcription factors (10). Sheet 11-12. Genes that are bound by HLX in K562 cells (11) or HL60 cells (12) and are deregulated in the RNA-seq in K562 cells (See also Supplementary Table 5). Sheet 13. Enriched regions for HLX in THP1 cells. Sheet 14-15. GO analysis (14) and IPA (15) in HLX bound genes in THP1 cells. Sheet 16-17. Motif analysis in THP1 cells before (16) or after masking of known transcription factors (17). Sheet 18. Enriched regions for H3K27ac in THP1 cells overexpressing HLX. Sheet 19-21. GREAT analysis for HLX bound regions in K562 (19), HL60 (20) and THP1 (21) cells

File Name: Supplementary Data 5
Description: RNA-seq K562, CD34+ cells. Sheet 1-2. Total RNA-seq results (1) and differentially expressed genes (2) between control and HLX KO K562 cells. Sheet 3-4. GO (3) and IPA (4) analysis of deregulated genes in HLX KO K562 cells. Sheet 5. Total RNA-seq results of differentially expressed genes between control and HLX KD CD34+ cells. Sheet 6. GO analysis of deregulated genes in HLX KD CD34+ cells. Sheet 7. Total RNA-seq results of differentially expressed genes between control and HLX OE CD34+ cells. Sheet 8. GO analysis of deregulated genes in HLX OE CD34+ cells.

File Name: Supplementary Data 6
Description: PPARD CHIP RNA-seq comparison. Sheet 1. Genes that are bound by PPARδ in publicly available datasets and are deregulated in CD34+ with overexpression or knockdown of HLX. Sheet 2-3. IPA analysis for genes bound by PPARδ and are deregulated in CD34+ overexpressing HLX (2) or CD34+ cells with HLX knockdown (3).
